# Effects of Low-Intensity Exercise on Exhaled Breath Condensate Biomarkers in Healthy Horses

**DOI:** 10.1021/acsomega.6c02766

**Published:** 2026-10-20

**Authors:** Thasla F. Santi, Bianca Barbosa, Bianca Faria Cuman, Giulia C. R. Pelle, Carolina R. Leitolles, Saulo H. Weber, Tiago M. Oliveira, Paulo M. Bogossian, Pedro V. Michelotto

**Affiliations:** † Graduate Program in Animal Science, Pontifícia Universidade Católica do Paraná (PUCPR), Rua Imaculada Conceição 1155, Prado Velho, 80215-901 Curitiba, Paraná, Brazil; ‡ Veterinary Medicine Program, Pontifícia Universidade Católica do Paraná (PUCPR), Rua Imaculada Conceição 1155, Prado Velho, 80215-901 Curitiba, Paraná, Brazil; § Department of Internal Medicine, 28133 School of Veterinary Medicine and Animal Science of the Universidade de São Paulo (FMVZ/USP), Avenida Professor Doutor Orlando Marques de Paiva 87, Butantã 05508-270 São Paulo-SP, Brazil; ∥ Veterinary Medicine Program, Universidade Municipal de São Caetano do Sul (USCS), Rua Santo Antônio 50, Centro, 09521-160 São Caetano do Sul, São Paulo-SP, Brazil

## Abstract

Exhaled breath condensate (EBC) has emerged as a promising diagnostic tool for equine respiratory disease. However, the influence of exercise on the biomarkers of EBC remains unclear. This study aimed to evaluate the influence of low-intensity exercise on EBC biomarkers (hydrogen peroxide, pH, and nitrite) in healthy horses and to assess the feasibility of postexercise sample collection under real-world conditions. Twenty clinically healthy horses (ten Quarter Horses and ten Brazilian Sport Horses) were evaluated. Respiratory rate (RR) and heart rate (HR) were recorded at rest, followed by EBC collection using a collector developed by our research group. Horses then performed 10 min of trotting, low-intensity protocol, after which RR, HR, and EBC samples were reassessed. Upper airway endoscopy and tracheal lavage with differential cytology confirmed respiratory health. Biomarker concentrations were analyzed by using appropriate parametric tests. Light exercise significantly increased RR, HR, and EBC sample volumes (*p* < 0.05). Sample volume evidenced a weak association with ambient temperature (*p* = 0.014, *r* = 0.186) and relative humidity (*p* = 0.010, *r* = 0.218). Exercise did not significantly affect pH (*p* = 0.380), hydrogen peroxide (*p* = 0.152), or nitrite (*p* = 0.218). Nitrite was negatively associated with ambient temperature (*p* = 0.023, *r* = −0.056) and relative humidity (*p* < 0.001, *r* = −0.428) and evidenced a moderate positive association with hydrogen peroxide in postexercise (*p* = 0.004, *r* = 0.611). Under the conditions evaluated, light exercise did not alter equine EBC biomarker concentrations, supporting the feasibility of postexercise sampling.

## Graphical abstract

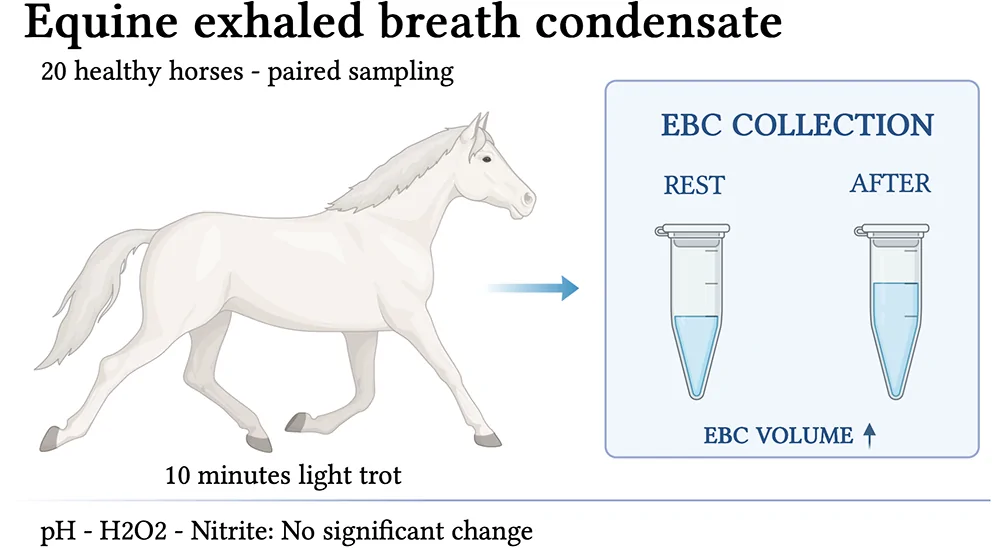

## 1 Introduction

The demand for the development of noninvasive, field-based diagnostic techniques, associated with the use of biomarkers to identify respiratory diseases in horses, has been increasingly recognized.^1^ In this context, the assessment of the airways through exhaled breath condensate (EBC) emerges as a promising noninvasive tool for the investigation and monitoring of respiratory disorders through the analysis of inflammatory biomarkers.^2–4^

However, the outcomes arising from the analysis of EBC are prone to be affected by uncontrolled variables, including environmental factors, physiological patterns of the patient, and even the type of equipment and collection technique.^4,5^ In this context, there is a need to discern the influence of exercise on the biomarkers measured from EBC collection.^6^

In the study conducted by Cathcart et al.,^2^ stabled racehorses without respiratory disease were evaluated before and after high-intensity exercise, with biomarker assessment performed after respiratory rate had returned to values close to preexercise levels. This study demonstrated a significant change in pure pH during the postexercise period; however, this finding was not influenced by ambient temperature, humidity, or respiratory rate. In studies conducted in humans, it is recommended that patients remain at rest for at least 1 h prior to EBC collection due to pH alterations.^7^ Similarly, Schleiss et al.^8^ observed that higher airflow is associated with increased H_2_O_2_ concentrations.

On the basis of these studies, the present work aimed to measure the biomarkers H_2_O_2_, pH, and nitrite in order to evaluate the potential influence of physical exercise on these parameters, as well as to assess the feasibility of collecting EBC samples from horses under real-world conditions, by analyzing biomarker variability before and after exercise.

## 2 Materials and Methods

### 2.1 Animals and Ethics

Twenty healthy horses from two breeding farms were included, comprising ten Quarter Horses (QH) and ten Brazilian Sport Horses (BSH), located in the municipalities of Almirante Tamandaré, Campina Grande do Sul and Campo Largo, respectively, in the state of Paraná, Brazil.

Animals included in the study had a mean age of 4.7 ± 1.8 years (range: 3–11) and were managed under a feeding regimen consisting of concentrate provided twice daily in individual stalls, in addition to hay and water ad libitum. The animals were maintained under stabled conditions and allowed turnout for 3 to 4 h daily. QH were undergoing intensive training for competitions, whereas the BSH were in the initial phase of training for show jumping and at the beginning of their competitive participation; however, at the time of evaluation, all animals were outside the competition period.

Ethical approval was obtained from the Institutional Animal Care and Use Committee of the Pontifical Catholic University of Paraná (PUCPR), under protocol number 4151131123.

### 2.2 Study Design

Samples were collected at rest and immediately after a light exercise. The exercise protocol consisted of light trotting the horse for 10 min over a circular sand arena. Heart rate (HR) was measured by auscultation using a stethoscope positioned between the third and sixth intercostal spaces for 1 min. Respiratory rate (RR) was obtained by auscultation of the midtrachea, simultaneously with counting thoracoabdominal movements. Measurements were performed with the animals at rest and immediately after completion of the 10 min exercise period.

After measurement of RR and HR, the animals underwent the first EBC collection at rest, remaining for 15 min with the collector positioned in one nostril. Subsequently, the animals were taken to a round pen, where they performed continuous trotting for 10 min, characterized as light exercise. At the end of the exercise, immediately after measurement of RR and HR, the second collection was performed (Figure 1), after which the animals returned to their stalls, an environment to which they were accustomed.

**Figure 1:**
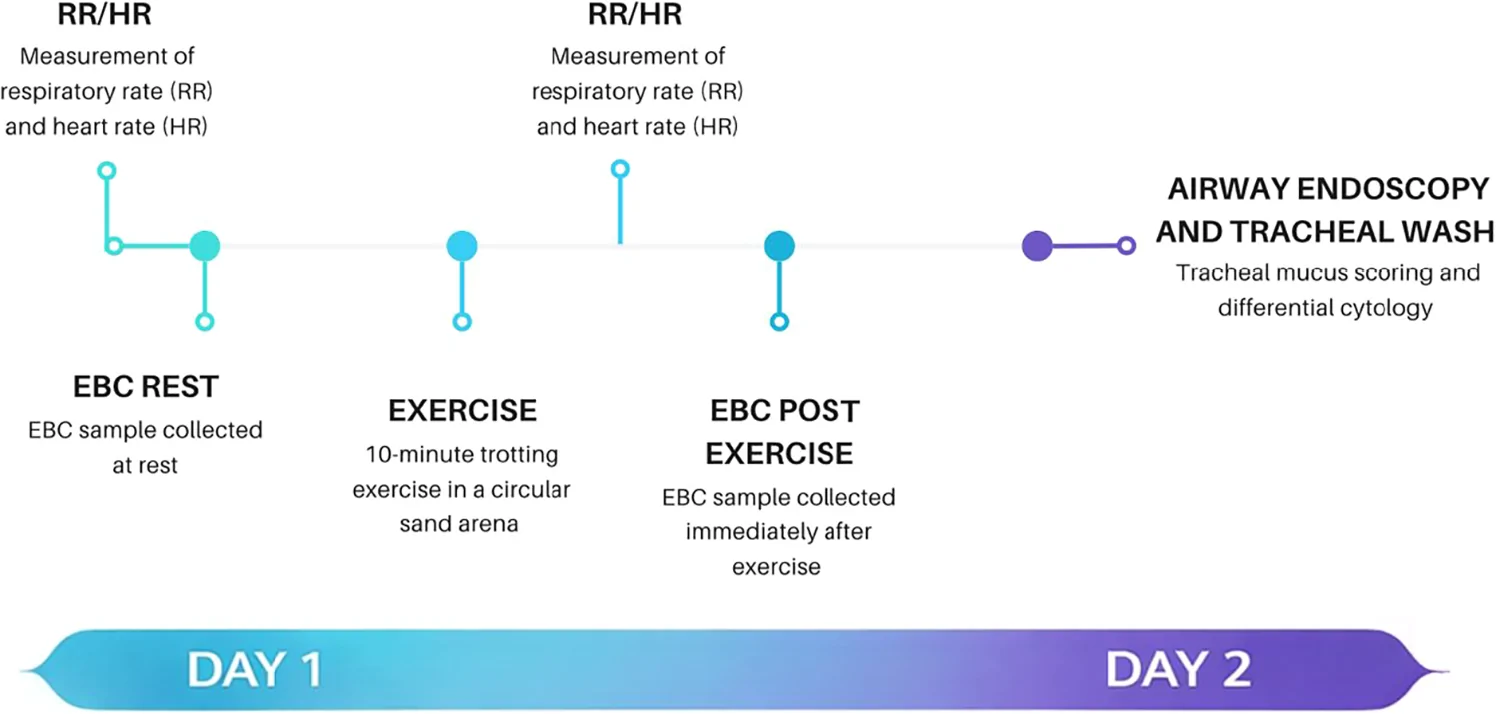
Timeline of the experimental protocol. Day 1 (resting and postexercise EBC collection with RR/HR measurements) and day 2 (airway endoscopy and tracheal wash).

The collected samples were immediately transferred to microcentrifuge tubes and kept under refrigerated conditions according to the requirements of each analysis. Concomitantly, ambient temperature and relative humidity were recorded using a thermo-hygrometer (Incooterm, São Paulo, Brazil) positioned near the animal throughout the entire collection period.

Exercise intensity was classified as low based on the characteristics of the protocol, consisting of 10 min of continuous trotting at a self-selected pace, without cantering or jumping efforts, and on the animals’ training history, as all horses were in regular training and routinely performed this type of work. Exercise intensity was not quantified using blood lactate concentration, percentage of maximal heart rate, or continuous workload monitoring.

### 2.3 EBC Collection Device

The device was 3D-printed using PETG filament with a 3D printer (CL2 Pro Plus–Cliever, Belo Horizonte, Brazil) and consists of a single condensation compartment measuring approximately 13 cm × 9 cm, with an internal cavity designed to hold water for condensate formation.^9^

For device preparation, the material was lined with an A4 plastic sheet to isolate the material obtained from each animal and prevent contamination.

For nasal cleaning, three dry gauze pads were gently rubbed from the alar fold to the nasal floor, with all procedures performed by the same handler. The device was then positioned in one nostril for 15 min, allowing condensation as warm exhaled air contacted the cold surface, after which the plastic lining was removed and the sample transferred to a sterile tube for sample storage.

### 2.4 Laboratory Analysis of Biomarkers

Samples were thawed at room temperature (23.3 °C) with a relative humidity of 56%, measured using a thermo-hygrometer (Laboratus–Fepro Mut60OS). pH measurement was performed using a benchtop pH meter (HI98103, Hanna Instruments, São Paulo, Brazil), as described by Duz et al.^10^ Hydrogen peroxide (H_2_O_2_) concentration was determined by spectrophotometry at 450 nm (VersaMAx Microplate Reader, Molecular Devices, San Jose, California, USA), with the Amplex Red Hydrogen Peroxide/Peroxidase Assay Kit (Molecular Probes Inc., Willow Creek Road, Eugene, Oregon, USA), following the manufacturer’s instructions. Each analysis used 50 μL of samples, and results were expressed in μmol/L. Nitrite concentration was measured using the Griess reagent, with spectrophotometric Reading at 548 nm, using 150 μL of samples for each analysis.

### 2.5 Airway Endoscopy and Tracheal Wash Collection, Processing, and Analysis

Airway endoscopy and tracheal wash sample collection were performed within the same week of the EBC collections, which were carried out before exercise on a rest day for the animals. The amount of mucus in the tracheal lumen was assessed using a scoring system ranging from 0 to 5.^11^ In addition, tracheal wash (TW) samples were collected using 20 mL of sterile saline solution. All materials were placed in a sterile silicone-coated container and refrigerated until processing of all samples, which occurred within a maximum period of 2 h after completion of the collections. After processing, slides were prepared for cytological analysis and stained using a Romanowsky-type stain (Panótico Rápido, Laborclin, Pinhais, Brazil). For cytological evaluation, 300 cells were counted.

Cytology was considered normal when it showed less than 20% neutrophils, more than 10% lymphocytes, and less than 1% eosinophils.^12^

### 2.6 Statistical Analysis

Values of the biomarkers pH, hydrogen peroxide (μmol/L), and nitrite are presented as mean ± standard deviation. Data normality was assessed using the D’Agostino–Pearson test.

Results of the analyses of EBC biomarkers at different time points (rest and exercise) were analyzed using a general linear model (GLM) for paired samples. Correlations between ambient temperature and relative humidity and the biomarkers were assessed using the Pearson correlation test.

Comparisons of biomarker values between breeds were performed using an independent samples Student’s *t* test, assuming unequal variances.

All statistical analyses were performed using IBM SPSS Statistics, version 25.0 for Windows (IBM Corporation, New York, USA), with *p* values of < 0.05 considered statistically significant.

## 3 Results

All horses were considered clinically healthy, presenting a tracheal mucus score of 0 on an endoscopic evaluation.

Exercise was associated with a significant increase in RR (*p* < 0.001), HR (*p* < 0.001), and sample volume obtained (*p* = 0.005). Sample volume showed a weak positive association with ambient temperature (*p* = 0.014, *r* = 0.186) and relative humidity (*p* = 0.010, *r* = 0.218) (Table 1).

**Table 1:** Values of Parameters Assessed during EBC at Rest and during Exercise in All Animals^a^

Parameters	Rest	Postexercise
Ambient temperature (°C)	19.7 ± 8.82 (12–26)	
Relative humidity (%)	61.8 ± 10.50 (46–78)	
Volume (μL)	1.10 ± 0.35 (0.7–1.9)	1.30 ± 0.24 (0.9–1.7)
Heart Rate (beats per minute)	38.70 ± 7.30 (24–59)	80.80 ± 10.53 (66–104)
Respiratory rate (respirations per minute)	17.90 ± 4.54 (8–24)	49.0 ± 11.48 (24–69)
pH	7.50 ± 0.45 (6.5–8.2)	7.63 ± 0.44 (6.7–8.4)
Hydrogen peroxide (μmol/L)	0.470 ± 0.422 (0.12–1.83)	0.665 ± 0.405 (0.19–1.56)
Nitrite	111.96 ± 68.79 (24.88–283.40)	136.36 ± 70.79 (29.16–312.51)

a °C, Celsius degree; %, percent; μL, microliters; μmol/L, micromoles per liter. Results are presented as mean ± standard deviation (minimum-maximum).

Conversely, exercise did not significantly affect the biomarkers evaluated in EBC, including pH (7.63 ± 0.44 vs 7.50 ± 0.45, *p* = 0.380), H_2_O_2_ (0.665 ± 0.406 vs 0.470 ± 0.422 μmol/L, *p* = 0.152), and nitrite (136.36 ± 70.79 vs 111.96 ± 68.79 absorbance at 548 nm, *p* = 0.218) (Table 1).

EBC nitrite concentration showed a negative association with ambient temperature (*p* = 0.023, *r* = −0.056) and relative humidity (*p* < 0.001, *r* = −0.428). In addition, nitrite showed a strong positive correlation with H_2_O_2_ concentration in the postexercise period (*p* = 0.004, *r* = 0.611).

The ambient temperature was moderately positively correlated with pH during exercise (*p* = 0.026, *r* = 0.494).

Results of the cytological evaluation, stratified by breed (Quarter Horse and Brazilian Sport Horse) and for the overall sample, are presented in Table 2.

**Table 2:** Cytological Data Values of Tracheal Wash Samples Obtained from Quarter Horses (*n* = 10) and Brazilian Sport Horses (*n* = 10) at Rest^a^

Cytology	Quarter Horse	Brazilian Sport Horse	Total
Macrophages (%)	69.50 ± 3.17 (66–77)	68.30 ± 2.76 (64–72)	68.90 ± 3.11 (64–77)
Neutrophils (%)	3.70 ± 0.78 (3–5)	2.20 ± 0.87 (1–4)	2.95 ± 1.15 (1–5)
Mast cells (%)	0.90 ± 0.70 (0–2)	1.00 ± 0.77 (0–2)	0.95 ± 0.76 (0–2)
Lymphocytes (%)	25.1 ± 3.94 (15–29)	28.2 ± 3.25 (23–34)	26.65 ± 4.03 (15–34)
Eosinophils (%)	0.8 ± 0.4 (0–1)	0.3 ± 0.46 (0–1)	0.55 ± 0.51 (0–1)

a %, percent. Results are presented as mean ± standard deviation (minimum-maximum).

Values of the EBC biomarkers evaluated in comparison between the breeds studied are presented in Table 3.

**Table 3:** Values of Biomarkers Investigated in EBC from Quarter Horses and Brazilian Sport Horses, Evaluated at Rest (R) and after Exercise (E)^a^

	Quarter Horse	Brazilian Sport Horse	*p* value
Volume R (μL)	1.31 ± 0.33 (0.9–1.9)	0.82 ± 0.11 (0.7–1.0)	0.001
Volume E (μL)	1.33 ± 0.23 (0.9–1.7)	1.33 ± 0.25 (1.0–1.7)	0.50
pH R	7.33 ± 0.44 (6.5–8.5)	7.66 ± 0.41 (6.8–8.2)	0.06
pH E	7.71 ± 0.58 (6.7–8.4)	7.54 ± 0.21 (7.2–7.8)	0.21
H_2_O_2_ R (μmol/L)	0.60 ± 0.56 (0.13–1.83)	0.35 ± 0.12 (0.19–1.56)	0.11
H_2_O_2_ E (μmol/L)	0.58 ± 0.30 (0.21–1.11)	0.75 ± 0.48 (0.19–1.56)	0.20
Nitrite R	83.45 ± 58.23 (24.88–208.63)	140.47 ± 66.70 (59.65–283.40)	0.04
Nitrite E	100.87 ± 43.52 (29.16–174.96)	171.86 ± 74.91 (49.65–312.51)	0.01

a μL, microliters; μmol/L, micromoles per liter. Results are presented as mean ± standard deviation (minimum-maximum). The *p* value refers to the comparison between breeds.

## 4 Discussion

The present study evaluated 20 healthy horses from two different breeds and sporting disciplines at rest and immediately after exercise, using a crossover design. The aim was to investigate whether this level of physical activity could be associated with variations in inflammation-related biomarkers in EBC based on samples collected under both conditions. The results demonstrated that the low-intensity exercise employed did not result in significant changes in pH, H_2_O_2_, and nitrite concentrations between the pre- and postexercise periods, although breed-related variations were observed.

Regarding the exercise component and pH values, Cathcart et al.^2^ also evaluated healthy horses undergoing exercise, however, at high intensity. The authors observed a significant increase in pure (nondeaerated) pH values after exercise (4.79 ± 0.59) compared with rest (4.51 ± 0.23). In our study, healthy horses undergoing/undertaking/sustaining low-intensity exercise showed no significant differences in EBC pH values, with postexercise values (7.63 ± 0.4) and resting values (7.50 ± 0.45). Although the protocols differed markedly in exercise intensity, both studies showed an increase in postexercise pH values, suggesting a common physiological response that appears to be influenced by workload and exercise duration.

These pH changes may be related to an increased airflow velocity and pulmonary blood flow during exercise, leading to greater production and delivery of solutes from the pulmonary lining fluid. This process may alter EBC pH and represent a physiological response to exercise, resulting from increased systemic production, particularly of ammonia in the airways,^2^ one of the exhaled volatile compounds.^13,14^ Ammonia is produced in the bronchial epithelium through the action of glutaminase and acts as an important buffer in the airways.^15^ However, ammonia production in the airways of horses has remained unknown. Nevertheless, increased environmental concentrations must also be considered, as this has been shown to be significantly associated with higher levels of exhaled ammonia.^16^ This factor might have influenced the results of this study, although this was not investigated, but the investigated animals used to spend most of their time in stalls, an environment in which ammonia is commonly produced through the degradation of urine, suggesting that this be included in next studies.

Another relevant point regarding pH variation concerns the cooling method used for EBC collection. Whittaker et al.^5^ compared different cooling methods and observed higher pure (nondeaerated) pH values in ice-cooled samples at 0 °C (6.87–7.49) compared with samples collected using dry ice at −75 °C (4.30–6.45) and ethanol slurry at −100 °C (5.84–6.26). In the study by Duz et al.,^10^ samples were collected using an ethanol slurry with liquid nitrogen, reaching −80 °C, and pH values in healthy horses ranged from 5.68 to 6.72. In our study, ice was used as the cooling method, as the device itself contains an integrated thermal chamber and is placed directly in a freezer.^9^

In addition, it is worth noting that pH is unstable outside the respiratory tract due to the volatility of CO_2_ molecules, which dissipate from the fluid and may lead to an increase in EBC pH.^17^ In the present study, argon deaeration was not performed, a technique recommended by some authors to minimize this variability and maintain pH stability. Nevertheless, our results did not differ from those obtained in studies that also measured pure pH, suggesting that the absence of the deaeration process did not compromise data interpretation. This is supported by our previous pilot study,^18^ in which samples were analyzed without deaeration and showed consistent results.

Regarding H_2_O_2_, it is a compound released by neutrophils, eosinophils, macrophages, and airway epithelial cells, playing a role in both defense against infectious agents and inflammatory signaling.^17^ In healthy horses, reported concentrations vary in the literature, with field values of approximately 1 μmol/L^3^, 0.4 ± 0.2 μmol/L^19^ and 0.1–0.8 μmol/L,^20^ and approximately 0.97 μmol/L maintained under field conditions,^18^ whereas horses housed in stalls exhibit levels close to 1.6 μmol/L^3^. In the present study, horses had daily access to pasture for three to four hours and showed a resting concentration of 0.470 μmol/L, which is consistent with values reported under field management conditions. These findings, together with the pH results, highlight the importance of access to pasture, even for limited periods, in maintaining a balanced respiratory environment.

The measurement of postexercise H_2_O_2_ concentration has not yet been described in horses. However, studies conducted in healthy, nonsmoking humans provide a comparative reference. In a cycling exercise protocol lasting 10, 30, and 90 min, Tuesta et al.^21^ observed an increase in H_2_O_2_ levels after 90 min of exercise compared with the values recorded after 30 min. Similar to what was observed in our study, Marek et al.^17^ also reported that 10 min of light exercise in healthy young individuals did not promote changes in H_2_O_2_ concentration in EBC, suggesting that exercise duration and intensity may determine peroxide concentration in EBC.

In the study by Mercken et al.,^22^ an increase in peroxide production in the EBC of healthy humans subjected to 12 min of maximal-intensity exercise was observed. However, when the same exercise duration was performed at approximately 40% of the maximal power, no changes in the measured values were observed. Considering these findings, we suggest that, in healthy animals already undergoing training under athletic conditions, such as those included in the present study, the exercise intensity employed may not have been sufficient to induce detectable changes in H_2_O_2_ concentrations.

Nitrite had not yet been measured in equine EBC, as previous studies have primarily focused on the evaluation of pH, H_2_O_2_, and, more recently, leukotriene B_4_. Nitrite, a stable end product of nitric oxide oxidative metabolism in aqueous media,^23^ has been widely used as a marker of airway inflammation in humans.^24,25^ During exercise, nitric oxide contributes to bronchodilatation and pulmonary vasodilatation.^26^ Accordingly, we evaluated nitrite concentrations in the EBC of healthy horses at rest and after light exercise, and no significant differences were observed between the sampling times. In contrast, increased EBC nitrite concentrations have been reported following prolonged exercise in humans, including amateur runners after long-distance races of 21 and 42 km,^27^ 10 km races in physically active individuals,^28^ and swimmers subjected to outdoor running.^29^ Tuesta et al.^21^ observed no significant changes in EBC nitrite concentrations following 10 or 30 min of cycling at 30% of VO_2_max, whereas an increase was detected following 90 min. These findings suggest that exercise duration may influence EBC nitrite responses even at a low intensity. However, direct comparisons are limited by differences in species, exercise protocols, and sampling timing; in the study by Tuesta et al., EBC was collected 80 min after exercise, whereas collection in the present study began shortly after the postexercise physiological measurements. Taken together, these studies suggest that the exercise duration and intensity may influence EBC nitrite concentrations. Under the conditions evaluated in the present study, no statistically significant changes in nitrite concentrations were detected following 10 min of trotting.

An additional consideration when interpreting EBC biomarker concentrations is the potential influence of the dilution effects. Respiratory droplets originating from the airway lining fluid become variably diluted by water vapor during exhalation and condensate formation, which may affect the measured concentration of nonvolatile biomarkers. In the present study, exercise significantly increased the respiratory rate and EBC sample volume, suggesting a greater contribution of condensed water vapor to the collected samples. Consequently, biomarker concentrations may not necessarily reflect proportional changes in the absolute amounts of compounds released from the airways. Therefore, the absence of significant differences in pH, H_2_O_2_, and nitrite concentrations between resting and postexercise samples should be interpreted with this limitation in mind. Dilution remains one of the major methodological challenges in EBC research, and no universally accepted dilution marker has yet been established.^4,30^ Previous studies have proposed approaches based on reference solutes and dilution correction strategies; however, further validation is still required before their routine application. Future studies should investigate dilution correction methods to improve the interpretation of EBC biomarker concentrations under different physiological and exercise conditions.

The present study was not primarily designed to investigate breed-related differences in EBC biomarkers; nevertheless, an exploratory comparison was performed. BSH exhibited higher nitrite concentrations than QH, both at rest and after exercise. These findings should be interpreted cautiously because of the limited sample size and the association of the breed with sporting discipline, training background, and farm environment. At rest, the lower sample volume obtained from BSH may have reflected less dilution and, consequently, higher nitrite concentrations;^4^ however, this possibility does not explain the postexercise difference, when sample volumes were similar between breeds. Differences in training background may also have contributed, as BSH were in the early stages of show-jumping training, whereas QH were undergoing intensive barrel-racing training. However, the influence of the training background on EBC nitrite concentrations was not directly assessed, and its contribution remains uncertain. Additionally, as each breed was housed at a different farm, environmental conditions may have contributed, particularly relative humidity, which was inversely associated with nitrite in the present study. Consequently, the observed differences cannot be attributed exclusively to the breed. Further studies comparing breeds under standardized exercise, training, and management conditions are needed to clarify these findings.

The negative association observed between the nitrite concentration and relative humidity highlights the potential influence of environmental conditions on EBC measurements. Relative humidity may affect condensate formation and the degree of dilution of nonvolatile biomarkers, thereby contributing to variability in measured concentrations. Similar effects have been reported in previous studies evaluating EBC biomarkers under exercise conditions, suggesting that environmental factors should be considered when interpreting respiratory biomarker data.^26^ Although the magnitude of this association was moderate, these findings reinforce the importance of recording and reporting environmental variables during the EBC collection. For future field-based studies, the standardization of collection conditions and consideration of ambient humidity may improve comparability between studies and facilitate the interpretation of EBC biomarkers as indicators of respiratory health and disease.

This study presents methodological limitations. Although the sample size was relatively small, which may limit the detection of minor biological effects, it is within the range commonly reported in equine EBC studies.^4^ Moreover, the absence of changes in EBC biomarkers is consistent with human studies in which short, low-intensity exercise did not alter EBC H_2_O_2_ concentrations,^17,22^ supporting the interpretation that the exercise protocol employed was not sufficient to modify these biomarkers. Nevertheless, larger studies are warranted to confirm these findings and to evaluate higher exercise intensities. Regarding the exercise protocol, optical sensor-based devices were not employed for objective and quantitative assessment of heart rate, respiratory rate, exercise speed, or workload during the trotting period. Likewise, physiological indicators commonly used to characterize exercise intensity, such as blood lactate concentration or percentage of maximal heart rate, were not evaluated. Heart rate and respiratory rate were measured using a stethoscope at rest and immediately after exercise, confirming significant increases relative to baseline and indicating a physiological response to exercise. Trot intensity was not predefined, and horses were allowed to maintain a comfortable, self-selected pace for 10 min. All animals were familiar with the round pen and accustomed to lunge work, which likely reduced the influence of handling-related stress on physiological parameters. Therefore, although a physiological response to exercise was confirmed, the classification of exercise intensity as low was based primarily on the characteristics of the protocol and the animals’ training history. Future studies incorporating standardized exercise protocols and objective workload measurements, including exercise speed, blood lactate concentration, and continuous physiological monitoring, are warranted to further elucidate the effects of exercise on EBC biomarker variability. Furthermore, only three EBC biomarkers were evaluated, and exclusively in healthy horses. Future studies including additional inflammatory biomarkers, such as leukotriene B_4_,^3^ and horses with naturally occurring respiratory disease, such as equine asthma,^1^ are needed to further establish the clinical applicability of postexercise EBC sampling.

The scarcity of studies evaluating the influence of exercise on EBC in horses still limits direct and in-depth comparisons with the available literature. Although human medicine has already explored this topic, controversies remain regarding the impact of exercise type, intensity, and duration, as well as the cooling method of the condenser. The findings of the present study indicate that light exercise did not significantly alter the evaluated biomarkers in BSH and QH engaged in show jumping and barrel racing, respectively. Under these conditions, it appears feasible to collect samples after exercise for the assessment of airway inflammation markers.

Additionally, recent advances in exhaled breath analysis have demonstrated the applicability of multimodal sensor arrays combined with artificial intelligence for evaluating physiological and clinical conditions, including those during exercise. These approaches range from CNN-based time-series analysis of breath sensor signals for metabolic and physiological monitoring^31^ to hierarchical deep learning frameworks for multicondition classification based on VOC patterns, with improved robustness and scalability,^32^ as well as multimodal sensor integration strategies.^33^ Although they show high diagnostic potential, these methods rely on gas-phase analysis and pattern recognition, differing fundamentally from exhaled breath condensate analysis, which enables direct biochemical biomarker assessment. Therefore, these approaches should be considered complementary rather than equivalent, and their integration remains an emerging field, particularly in the field of veterinary medicine.

## 5 Conclusion

Under the conditions evaluated, 10 min of light exercise did not affect the concentrations of the pH, nitrites, and H_2_O_2_ in equine EBC, supporting the feasibility of sample collection following low-intensity exercise, as this results in greater sample volume. Further understanding of the respiratory response to exercise still requires additional studies involving different sporting disciplines as well as varying exercise intensities and durations. We suggest that future investigations should determine whether postexercise sampling can be extended to real competition or training settings, thereby enhancing the practical applicability of the technique in differentiating healthy horses from those presenting airway diseases.
